# Enlargement of the Tonsils—III

**Published:** 1908-10-31

**Authors:** 


### Laryngology and Rhinology.

### ENLARGEMENT OF THE TONSILS—III.

TREATMENT (*continued.*)

Excision remains the only treatment of value in most cases. Very various methods have been employed, which resolve themselves into three distinct procedures: removal with the guillotine, with punch-forceps ("morcellement"), and enucleation. Removal with the guillotine still remains the foremost method of treatment; it is true that the entire base of the tonsil is not removed, and that, if performed unskilfully or in unsuitable cases, the operation may be followed by recurrence; but it nevertheless gives entirely satisfactory results in the large majority of cases of simple parenchymatous enlargement in children and young adults. The operation may be performed on adults under local anæsthesia, but a general anæsthetic is preferable for children and nervous adults. In this case the patient should lie in the right lateral position with the head low; a Mackenzie's guillotine or some modification of this is advised, the left forefinger is placed on the tonsil and the instrument, passed between finger and tonsil, is fitted over the projecting mass. It should be passed in a somewhat downward direction and insinuated over the pedunculated lower end of the gland; the handle is then brought to the opposite angle of the mouth so as to press the distal end of the guillotine well outwards behind the tonsil, and the knife is worked with the left hand, while the anæsthetist makes inward pressure behind the angle of the jaw. All this can readily be done guided only by the sense of touch, but examination should then be made in a good light to see that the removal is complete; in cases of flat adherent tonsils punch-forceps may be used to complete the operation. A tonsil buried beneath an adherent anterior pillar cannot be engaged in the guillotine, but if the pillar be first dissected off it can generally be satisfactorily excised; the dissection may be done with a pair of scissors sharply curved on the flat or with a probe-pointed knife set at a right angle on its shaft. Enucleation is not necessary in the majority of cases, but it remains the best method of removing those flat tonsils, the seat of chronic lacunar tonsillitis, and especially the iregular fibrosed tonsils of older patients which are subject to recurring attacks of inflammation; in the latter case recurrent hæmorrhage is less likely to occur after enucleation, as the section passes beneath the cicatricial tissue, which prevents efficient retraction of the vessels. An anæsthetic is necessary, and the patient is placed on the side. No elaborate surgical armamentarium is required, for the entire operation can be done with a pair of curved scissors, a broad-toothed volsellum, and the finger; a Hartmann's punch-forceps, too blunt to cut through the substance of the tonsil, acts well as a volsellum. The tonsil is seized and raised from its bed, and the mucous membrane between the tonsil and anterior pillar is divided; the latter should not be cut, for it bleeds freely and its section causes unnecessary damage. The finger or the closed scissors is introduced into the supra-tonsillar fossa, and the gland separated downwards from the pharyngeal aponeurosis; a few snips with the scissors may be needed in places, and the separation is completed below with scissors or with a strong snare. Hæmorrhage is seldom very profuse and is controlled by sponging. It is convenient to have an assistant to sponge and to control the tongue with a depressor. The wound heals as readily as after the guillotine operation.

